# Neuroforaminal Stenosis in the Lumbosacral Spine: A Scoping Review of Pathophysiology, Clinical Manifestations, Diagnostic Imaging, and Treatment

**DOI:** 10.51894/001c.87848

**Published:** 2023-12-05

**Authors:** Daniel R. Cavazos, Devan O. Higginbotham, Fong Nham, Tannor Court, Scott McCarty, Anil Sethi, Rahul Vaidya

**Affiliations:** 1 Orthopaedic Surgery Detroit Medical Center; 2 Orthopaedic Surgery Wayne State University School of Medicine

**Keywords:** Neuroforaminal stenosis, lumbosacral, spine, review, stenosis, failed back surgery

## Abstract

**OBJECTIVE:**

To conduct the first scoping review of lumbosacral neuroforaminal stenosis with respect to the pathophysiology, symptomatic manifestations, diagnostic imaging, and treatment options.

**METHODS:**

A scoping literature review was conducted in accordance with the recommendations set forth by the Preferred Reporting Items for Systematic Reviews and Meta-Analyses (PRISMA), with English language restrictions stipulated to include articles pertaining to lumbosacral neuroforaminal stenosis. Databases maintained by PubMed, National Library of Medicine, Cochrane Central Register of Controlled Trials (Ovid), Scopus (Elsevier), Web of Science (Thomson Reuters), and Google Scholar were queried from their inception date through December 2022.

**SUMMARY OF THE EVIDENCE:**

A total of 276 articles were reviewed and 29 articles were included within the study. Within these articles, the anatomic origins of neuroforaminal stenosis were reviewed in detail and the resulting clinical manifestations were discussed. Recent studies evaluating the efficacy of existing diagnostic imaging modalities were summarized, along with potential future methods to improve sensitivity for detecting this entity and measuring foraminal stenosis via novel imaging techniques. Based on the literature, the conservative management and surgical treatment of lumbosacral foraminal stenosis were discussed.

**CONCLUSIONS:**

Lumbar neuroforaminal stenosis represents a significant source of radicular pain that is often compounded by delayed diagnosis and incomplete treatment. This article represents the first scoping review of lumbosacral neuroforaminal stenosis with focus on diagnosis, management, and treatment for associated radicular pain. The goal is to reduce the incidence of untreated or unrecognized neuroforaminal stenosis in the setting of a complex decompression and fusion, as well as to promote minimally invasive surgery to address radicular pain from neuroforaminal stenosis. Recent advances in diagnostic imaging and surgical techniques have the potential to improve the timeliness and durability of patients’ treatment options. Future directions for the diagnostic imaging of foraminal stenosis include efforts aimed at developing the nascent field of computerized mapping to reliably quantify stenosis and its impact on the exiting nerve root and associated dorsal root ganglia.

## INTRODUCTION

Orthopaedic spine surgeons continue to rely on surgical intervention to achieve precise decompression of neural elements in the lumbosacral spine, as it remains one of the most reliable operative treatment options. Stenosis of the lumbosacral spine is categorized as either central or lateral. Central spinal stenosis involves the area between the facet joints, involving the dural sac and its contents. The lateral zone is split into three zones: the lateral recess, foraminal space, and extraforaminal space. The lateral recess extends from the lateral edge of the dura to the medial edge of the pedicle. The foraminal space is the area bounded by the pedicles and the extraforaminal space is just distal to this space.^1^ Foraminal stenosis is characterized by the compression of the exiting nerve root in the foraminal space of the lumbosacral spine. Compression of the nerve root in this space causes radicular pain in that particular nerve root’s distribution.^1^

Neuroforaminal stenosis is a significant cause of debilitating pain due to lumbosacral radiculopathy.^2^ Inadequate decompression is one of the primary causes for early reoperation following lumbar decompressive procedures,^3^ but failure to resolve neural impingement within the foramina often represents an overlooked culprit for recalcitrant pain despite surgery. Just as a central disc herniation most readily encroaches upon the traversing nerve root, foraminal stenosis severely affects the exiting root at each lumbar motion segment.^1^

A traditional decompression involves removal of the anatomy causing compression to the central stenosis region and its associated dural sac and traversing nerve root. A foraminotomy is the decompression of the lateral stenosis regions affecting the exiting nerve root.^1^ Achieving the optimal foraminotomy requires striking a delicate balance between performing a thorough decompression (thus preventing residual stenosis) and facet joint preservation (thus avoiding iatrogenic instability). If foraminal decompression can be accomplished without exacerbating dynamic instability at the operative motion segment, fusion procedure can be avoided.^1^

The rationale behind this is to explore modalities that have the potential to avoid “the failed back surgery.” At an 8-10 year follow-up, the surgical treatment success for lumbar spinal stenosis was reported as 55% compared to 49% in conservative treatment cases in the Maine Lumbar Spine Study.^4^ The most common reasons for the failed back surgery include residual or recurrent herniation, undertreated or ignored foraminal stenosis, facet causes, paraspinal musculature scarring, or the battered nerve root.^2^ Foraminal stenosis is stated to be the culprit in 60% of failed back surgeries.^2,4^ So, untreated, undertreated, or ignored foraminal stenosis is accounting for a significant fraction of recurrent or residual pain in patients undergoing spine surgery for lumbosacral stenosis.

This paper aims to concisely outline the pathophysiology, clinical manifestations, diagnostic modalities, and treatment regimens for neuroforaminal stenosis of the lumbosacral spine. There is a current clinical gap in recognizing the clinical significance of foraminal stenosis in the treatment of stenosis. The goal of this clinical review article is to synthesize information on the pathophysiology, clinical manifestations, diagnosis, and treatment of foraminal stenosis to improve treatment outcomes for patients undergoing surgery for lumbosacral stenosis.

## METHODS

To perform a scoping review of all relevant primary studies, a comprehensive literature search was conducted according to the Preferred Reporting Items for Systematic Reviews and Meta-Analyses (PRISMA); the guidelines and recommendations for selecting qualified studies, assessing their methodological quality, and extracting the requisite data.^5^ All steps of the PRISMA process were carried out by authors F.N. and T.C. Keyword queries were conducted using Boolean operators to alternately link “lumbar foraminal stenosis” with either “diagnosis” or “treatment” to search the following databases: PubMed, MEDLINE (National Library of Medicine), Cochrane Central Register of Controlled Trials (Ovid), Scopus (Elsevier), Embase (Elsevier), CINAHL (EBSCO), Web of Science (Thomson Reuters), and Google Scholar from their inception dates through December 1, 2022, with no language restrictions stipulated. Since the keyword queries yielded limited results from the Cochrane Central Register of Controlled Trials, traditional medical subject heading (MeSH) searches were also performed, using the combination of “spinal stenosis” and “foramen.” The abstracts were reviewed by F.N. and T.C. to further characterize all the studies identified through this search strategy. To avoid any possibility of omissions, references cited by the retrieved studies were also independently evaluated. The exhaustive search of additional databases supplementary to PubMed yielded only three additional studies that ultimately qualified for inclusion. This is consistent with recent studies indicating that data sources beyond PubMed have a modest effect on the results of literature reviews.^6^

The inclusion criteria for studies to be included within this review included, original publications that included information about lumbosacral neuroforaminal stenosis which included either anatomical background, cadaveric analyses, pathophysiology, diagnostic studies and/or diagnostic interpretations, surgical and/or non-surgical interventions, and/or treatment outcomes. Exclusion criteria included non-original peer reviewed articles, articles without results and/or analyzed data, and articles not within the English language. Articles were initially screened in both their titles and abstracts, if they did not meet the inclusion criteria they were omitted from the review.

## SUMMARY OF THE EVIDENCE

### Search Results

**Figure 1.**
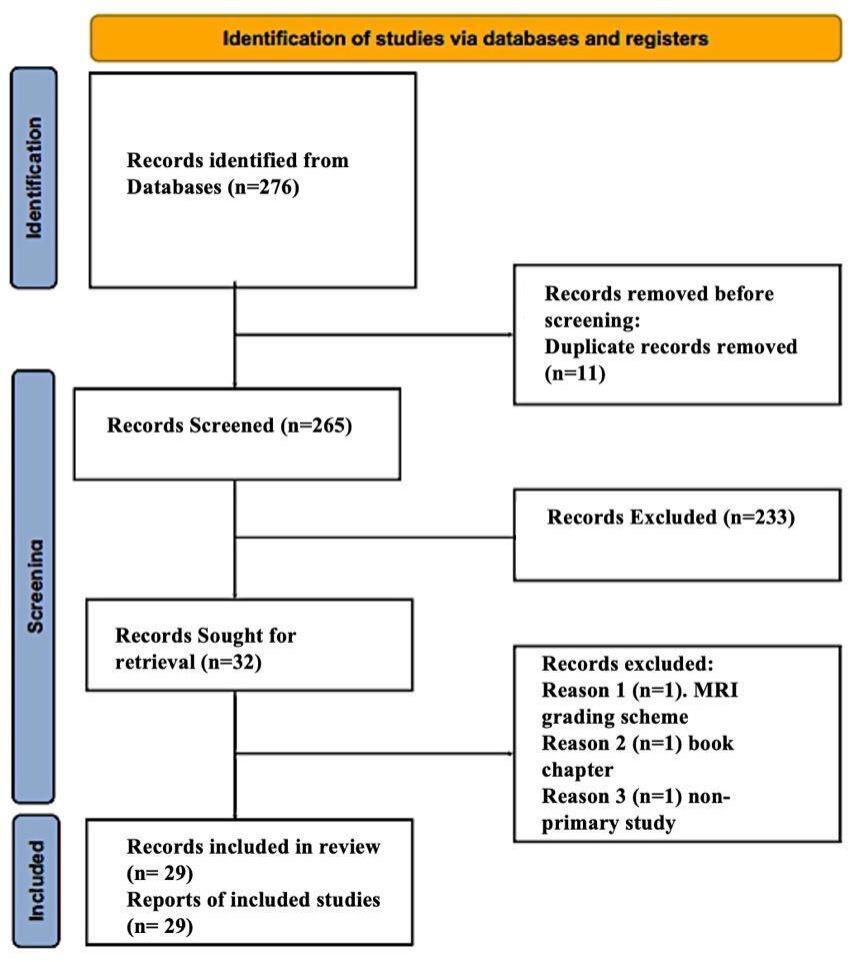
Preferred Reporting Items for Systematic Reviews and Meta-Analyses (PRISMA) article selection flowchart.

A total of 276 initial studies were identified, which were then narrowed down to 32 studies that were completely screened for inclusion. An additional 3 studies were excluded upon complete review, with comments for exclusion. Ultimately a total of 29 articles were included using the PRISMA process (Figure 1). The 29 studies are outlined by their primary aim, study design, and contribution to the scoping review in Table 1.

**Table 1.** Breakdown of the 29 studies used by their primary aim, study design, and contribution to the scoping review.

**Reference**	**Authors**	**Primary Aim**	**Study Design**	**Contribution**
^1^	Lee et al 2015	Lumbar Stenosis Review	Literature Review	Treatment
^2^	Yeung et al 2014	Review of Endoscopy in Foraminal Stenosis	Retrospective Cohort	Treatment
^3^	Shimizu et al 2016	30 Day Reoperation Causes in Spine	Retrospective Cohort	Background
^4^	Lurie et al 2015	Long-term Outcomes in Stenosis Surgery	Randomized Trial	Treatment
^5^	Moher et al 2009	PRISMA Review	Review	Methods
^6^	Halladay et al 2015	Data Sources in Systematic Reviews	Review	Methods
^7^	Jenis et al 2000	Review Lumbar Foraminal Stenosis	Review	Pathophysiology
^8^	Mandell et al 2017	Transforaminal Epidural Steroid Injection Review	Review	Pathophysiology
^9^	Hasegawa et al 1995	Anatomy of Lumbar Foramen	Cadaver Study	Pathophysiology
^10^	Orita et al 2016	Review Lumbar Foraminal Stenosis	Review	Pathophysiology
^11^	Zhao et al 2016	Anatomy of L5-S1 Foramen	Cadaver Study	Pathophysiology
^12^	Murata et al 2015	Vacuum Phenomenon and Foraminal Stenosis	Retrospective Cohort	Clinical Manifestations
^13^	Singh et al 2013	Dynamic Lumbar Foraminal Stenosis	Retrospective Cohort	Clinical Manifestations
^14^	Ren et al 2017	Dynamic Lumbar Foraminal Stenosis	Retrospective Cohort	Clinical Manifestations
^15^	Orita et al 2016	Neuropathic Pain in the Lumbar Spine	Retrospective Cohort	Clinical Manifestations
^16^	Porter et al 1984	Root Entrapment Syndrome Review	Retrospective Cohort	Clinical Manifestations
^17^	Yamada et al 2014	Leg Pain and Foraminal Stenosis Association	Retrospective Cohort	Clinical Manifestations
^18^	Shimizu et al 2020	Indirect Decompression in Stenosis	Retrospective Case Series	Clinical Manifestations
^19^	Cramer et al 2003	Dimensions of Lumbar Foramen MRI	Anatomic Study	Diagnostic Imaging
^20^	Senoo et al 2014	Dimensions of Lumbar Foramen 3D CT	Anatomic Study	Diagnostic Imaging
^21^	Lee et al 2010	Foraminal Stenosis Grading System on MRI	Retrospective Case Series	Diagnostic Imaging
^22^	Park et al 2012	Foraminal Stenosis Grading System on MRI	Retrospective Case Series	Diagnostic Imaging
^23^	Aota et al 2007	MRI and myelography for Diagnosis of Stenosis	Retrospective Case Series	Diagnostic Imaging
^24^	Yamada et al 2015	3D MRI Analysis of Stenosis	Retrospective Case Series	Diagnostic Imaging
^25^	Nemoto et al 2014	3D MRI Analysis of Stenosis	Retrospective Case Series	Diagnostic Imaging
^26^	Yamada et al 2017	3D MRI Analysis of Stenosis	Retrospective Case Series	Diagnostic Imaging
^27^	Eguchi et al 2016	Diffusion Tensor Imaging and Stenosis Types	Retrospective Case Series	Diagnostic Imaging
^28^	Kanamoto et al 2016	Diffusion Tensor Imaging and Double Crush	Prospective Study	Diagnostic Imaging
^29^	Genevay et al 2010	Lumbar Spinal Stenosis	Review	Treatment

### Pathophysiology

The literature review yielded five studies which contributed to the pathophysiology section. Three of the studies were reviews^7,8,10^ and two of them were cadaver studies.^9,11^ The neuroforaminal space is defined, in the coronal plane, by the medial and lateral fringes of the cephalad and caudal pedicles. In the sagittal plane, it is similarly defined as the distance between the superior and inferior borders of successive pedicles. This interpedicular distance represents the available area for the corresponding nerve root to occupy after leaving the thecal sac. Therefore, foraminal stenosis may result from any combination of distinct anatomical elements that impinge upon this defined osseous conduit as it is traversed by the exiting nerve root.^7^

Given the complexity of the neuroforamina and adjacent structures, a combination of factors contribute to foraminal stenosis. The most common causes include decrease in the adjacent disc height, impinging facet joint synovial cysts, redundant facet joint capsule, facet arthropathy, ligamentum flavum hypertrophy, degenerative or iatrogenic anterolisthesis, and osteophytes. The lumbar vertebrae are the largest of the vertebral spine, with comparatively short pedicles that impart an inverted tear drop configuration to the intervertebral foramina. This dramatically reduces the sagittal cross-section of the retrodiscal portion of the neuroforamen compared to the subpedicular notch. The retrodiscal division of the neuroforamen carries the exiting root, while the subpedicular notch division contains the neurovascular bundle.^7^

Each lumbar nerve root, with its ventral and dorsal components, leaves the thecal sac and intimately follows the inferomedial aspect of the pedicle, passing through the subpedicular notch that occupies a large portion of the canal. Previous studies have reported that nearly half of the available neuroforaminal space is accounted for by surrounding fat and radicular vessels.^7^ The retrodiscal portion is much narrower and sometimes obliterated, especially at L4-L5 level. This narrow retrodiscal space is due to the posterior attachment of the intervertebral disc to the superior portion of the inferior pedicle that causes a natural bulging behavior. The facet joint capsule and ligamentum flavum also narrow the retrodiscal space. This is in part due to the broad insertion of the ligamentum flavum to the anterior surface of the superior articular process that frequently extends to the superior portion of the pedicle.^8^ Normal lumbosacral foraminal dimensions are 20-23 mm in height and 8-10 mm in width. Less than 15 mm in height or less than 4 mm of posterior disc height are indicative of severe nerve root compression.^9^

Intervertebral disc degeneration causes a reduction in disc space height, followed by facet joint capsule and ligamentous laxity, resulting in dynamic instability. Progressive anterolisthesis induces superior and posterior subluxation of the superior articular processes of the more caudal vertebrae, manifesting as direct impingement of the neuroforamina. Dynamic instability also fosters accelerated facet arthropathy, with accompanying osteophyte formation, synovial cyst development, joint capsule hypertrophy, and ligamentum flavum thickening. Together, these factors contribute to central, subarticular recess, and neuroforaminal narrowing.^10^

Degenerative disc disease exacerbates the process of neuroforaminal narrowing due to accelerated osteophyte formation on the posterolateral margins of the vertebral bodies and herniation of the nucleus pulposus, both of which contribute to neuroforaminal stenosis. Posterolateral marginal osteophytes are most seen at L5-S1 level arising from the dorsolateral aspect of L5 and are believed to arise from the dissociation of Sharpey’s fibers from the vertebral endplates.^11^ The loss of disc height additionally reduces the vertical distance between pedicles. The pathophysiology and natural history of foraminal stenosis is well understood. Current research is aimed in understanding its role in clinical manifestations of refractory pain after surgery and adjacent segment disease.^1^

### Clinical Manifestations

The literature review yielded seven studies which contributed to the clinical manifestations section. Six of the studies were retrospective cohort studies^12–17^ and one of studies was a retrospective case series.^18^ Foraminal stenosis is a dynamic anatomic entity that is exacerbated by lumbar extension, particularly in the presence of vertebral anterolisthesis or vacuum disc phenomena,^12^ which reduces the cross-sectional area of the involved foramen by 30% and intensifies the mechanical encroachment upon the exiting nerve root.^13,14^ While the radiculopathy associated with foraminal stenosis predominantly results in appendicular pain with a dermatomal distribution, most patients experience axial low back pain, which is typically localized in the gluteal region.^15^ Concurrent clinical manifestations with a specific nerve root distribution include focal motor weakness, diminished sensation, or absent reflexes.^16^

Foraminal stenosis is best characterized by leg pain at rest, which is exacerbated by assuming supine or sitting positions, as well as the lateral decubitus position on the affected side.^17^ This is in stark contrast to the findings observed in cases of central disc herniation or canal stenosis, where pain is exacerbated by lumbar flexion and accompanied with nerve root tension signs such as straight leg raise or Laségue’s sign. In the presence of foraminal stenosis, additional neural compression zones may originate from a hypertrophic pars defect, laterally from the subarticular recess, or even extraforaminal in location. A misdiagnosis or inadequate treatment of the underlying pathology can increase the duration of foraminal stenosis symptoms, with the mean duration of low back pain and radicular leg symptoms being 43.7 and 15.3 months, respectively.^7^ The clinical gap in foraminal stenosis lies largely with the clinician in including it in the differential as a cause of radicular pain or persistent back pain in the failed back surgery.^2,4^

### Diagnostic Imaging

The literature review yielded ten studies which contributed to the diagnostic imaging section. Seven of the studies were retrospective case series,^21–27^ two were anatomic studies,^19,20^ and one of them was a prospective study.^28^ Several radiologic modalities, such as conventional radiographs, computed tomography (CT) and magnetic resonance imaging (MRI), can be used to delineate foraminal stenosis, with novel MRI techniques providing the most comprehensive evaluation of neural encroachment.^18^ Using extensive cadaveric studies, Hasegawa and colleagues quantified the relevant dimensions indicative of foraminal stenosis, suggesting that a posterior intervertebral disc height of less than 4 mm and a foraminal height less than 15 mm are consistent with clinical stenosis. However, the entire clinical context must be taken into account when examining each patient’s advanced imaging modalities.^9^ Cramer et al.^19^ evaluated the intervertebral foramen with MRI in 95 normal patients and found the suggested threshold of 15 mm to be reasonable for L1-L5 lumbar foraminal stenosis. Nonetheless, these investigators suggested a lower threshold for the L5-S1 interface, as these neuroforamina were found to be shorter than those at more proximal levels and a few asymptomatic elderly patients were found to have foraminal heights well below 15 mm at the L5-S1 level.^20^

Plain radiographs provide limited evaluation of the nonosseous structures of the spine. Nevertheless, they remain advantageous during the initial clinical evaluation to delineate global sagittal alignment, disc height, and foraminal anatomy. Compared to static CT or MRI studies, dynamic studies are more useful for identifying segmental instability or viewing the patient in lumbar extension, which invariably results in further narrowing of the neuroforamina.^1^ CT scans capture finer bony detail compared to plain films and allow for a more accurate evaluation of the neuroforamina.^1^ This imaging modality evaluates the overall anatomy of the foraminal space and identifies whether any spurs from the vertebral body or facet joints are impinging upon the foramen.^21^

**Figure 2.**
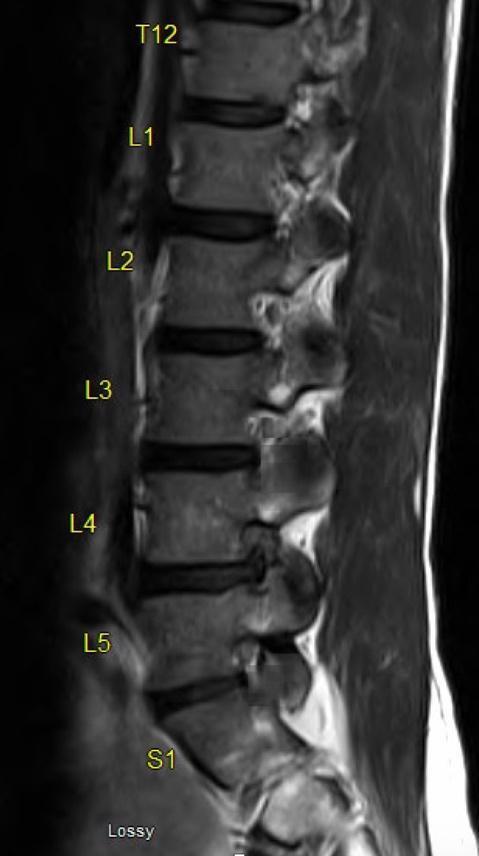
Figure 2 shows a T2 sagittal MRI of the lumbar spine with grade 0 or absence of stenosis at L2-3, grade 1 stenosis or mild foraminal stenosis at L3-4 with some fat obliteration in both the vertical and transverse directions, grade 2 stenosis or moderate foraminal stenosis at L5-S1 with perineural fat obliteration in all four directions without morphologic change in the nerve root, and grade 3 stenosis or severe foraminal stenosis at L4-5 showing complete perineural fat obliteration with nerve root collapse or deformation.

The lack of widely accepted diagnostic criteria or a grading system for describing MRI findings has made it more difficult to communicate the extent of foraminal stenosis. A landmark study described and evaluated a novel foraminal grading system using MRI and demonstrated near-perfect interobserver and intraobserver agreement. This grading system has further been found to correlate well with patients’ symptomatology and neurologic signs in a related study.^22^ On the basis of sagittal MRI cuts, the study characterized four grades of stenosis by focusing on both perineural fat obliteration and nerve root morphology.^21^ According to their classification system, grade 0 indicates the absence of foraminal stenosis; grade 1 indicates mild foraminal stenosis with perineural fat obliteration in two orthogonal directions, vertical or transverse; grade 2 refers to moderate foraminal stenosis with perineural fat obliteration in four directions without morphologic change to the nerve root; and grade 3 indicates severe foraminal stenosis with nerve root collapse or morphologic change (Figure 2).

MRI remains the imaging modality of choice for evaluating neuroforaminal stenosis. In general, stenosis is suspected when T1-weighted images show limited perineural fat surrounding the nerve root. Using conventional MRI to detect foraminal stenosis is limited by its inherent low resolution, given the dimensions of the lumbosacral neuroforamina, and its inability to depict the entire course of the nerve because of the slice gap in the captured images. Clinicians must also consider the high frequency of false positive results. Aota et al. calculated the efficacy of conventional MRI for diagnosing symptomatic foraminal stenosis, reporting the sensitivity, specificity, positive predictive value, and negative predictive value of MRI in this clinical setting to be 96%, 67%, 4%, and 100%, respectively.^23^

The advent of novel MRI-based imaging techniques has been able to circumvent some of the problems encountered by conventional MRI algorithms. A recent study compared the ability of three-dimensional (3D) MRI with conventional two-dimensional (2D) MRI in detecting foraminal stenosis. The 3D technique enables direct visualization of the root within the foramen, achieving significantly better intra- and inter-observer reliability as well as enhanced sensitivity for detecting stenotic lesions right away.^24^ Nemoto et al. used a 3D MRI algorithm with steady-state acquisition to show the efficacy of detecting neuroforaminal stenosis compared to standard 2D imaging.^25^ This technique uses reconstructed images without slice gaps to achieve superior spatial resolution with optimized signal-to-noise ratios, allowing for the visualization of the nerve roots in their entirety. In addition, this technique enables high-fidelity coronal reconstruction of neuroforaminal images, which are unavailable with conventional MRI when assessing for stenosis but have superior diagnostic performance compared to sagittal or axial images alone.^26^

Diffusion-weighted tensor imaging is a novel MRI protocol that is still in development. It evaluates the propensity and direction of water molecule diffusion to obtain intricate details about the course of nerve roots through the neuroforamina. More importantly, it provides quantitative tractograms,^27^ which have the potential to achieve a quantum leap in diagnosing foraminal stenosis to address the gap in misdiagnosis and undertreatment of foraminal stenosis in patients.^28^

### Treatment

The literature review yielded four studies which contributed to the treatment section. Two of the studies were reviews,^1,29^ one of them was a retrospective cohort study,^2^ and one of them was a randomized trial.^4^ The initial treatment of lumbar radicular pain, regardless of cause, consists of conservative measures such as nonsteroidal anti-inflammatory drugs, physical activity modifications, physical therapy, and transforaminal epidural corticosteroid injections. When the diagnosis is uncertain, selective nerve root blocks can provide a clearer clinical picture and are particularly useful because they can function as both a diagnostic and therapeutic tool.^1^

Patients with persistent pain despite conservative treatment and symptoms correlating with a compressive lesion identified on prior imaging are candidates for an elective neuroforaminal decompression procedure.^4^ A concomitant arthrodesis procedure may also be considered in those patients who exhibit stenosis in the setting of instability or deformity that results in sagittal or coronal imbalance.^1^

Decompression of lumbosacral foraminal stenosis can be performed through a midline or a muscle splitting Wiltse approach. With the midline approach, an incision is made longitudinally over the spinous processes at the appropriate level. The paraspinal muscles are elevated subperiosteally, and dissection is performed along the spinous process and lamina until reaching the relevant facet joint. This approach is ideal when other concomitant stenosis is present because it allows access to the central canal, lateral recess, and foramen. By performing a laminotomy, an undercutting medial facetectomy, and a dedicated foraminotomy, decompression will be achieved in all these zones. In addressing the foramina in particular, all culprits (e.g., epidural adipose tissue or incarcerated posterior longitudinal ligament) for neural compression are removed, exposing the underlying nerve root as it leaves the thecal sac. The surgeon must be careful to avoid removing >50% of either of the two facet joints for any single motion segment, as this can cause iatrogenic spondylolisthesis after the decompression, necessitating a subsequent fusion procedure to regain spine stability.^29^

When dealing with pure foraminal stenosis, the muscle splitting Wiltse approach may be employed. This approach entails an incision 3 cm lateral to the midline and the use of an interval between the multifidus and longissimus muscles to access the pars and transverse process, which are partially resected to allow for decompression of the foramen. In addition to these open approaches, the foramina can be accessed in a minimally invasive fashion via a tubular retractor to decompress the foramen.^2^ Minimally invasive approaches through tubular retractors avoids extensive paraspinal musculature dissection which can lead to postoperative scarring and residual pain. The transforaminal endoscope can be docked at Kambin’s safe triangle. This triangle also referred to as the “hidden zone,” is the right triangle over the dorsolateral disc. The triangle’s borders are defined by the exiting nerve root as the hypotenuse, the base as the endplate of the caudal vertebrae, and the height of the traversing nerve root or lateral border of the facet joint.^2^ This minimally invasive endoscopic technique allows for decompression of the exiting nerve root in the neuroforamen in addition to the traversing nerve root with minimal bony resection of the ventral aspect of the superior articular process to avoid destabilizing the spine.^2^

When concomitant instability or resection of the discosteophyte complex prevents adequate decompression, a fusion procedure may be necessary. Fusion procedures can indirectly decompress the neuroforamina at each motion segment by restoring the height of the intervertebral space and maintaining the local lordotic curvature of the spine.^29^ These outlined treatment modalities are well understood in spine surgery. The current clinical gap this article hopes to address is to help the clinician diagnose and recognize foraminal stenosis as a major pain generator and failure in spine surgery to optimize patient outcomes.^2,4^

### Strengths and Limitations

The merit of this paper resides in the fact that it is the first scoping review of lumbosacral neuroforaminal stenosis diagnosis and management in the literature. This will contribute to improved patient care through better understanding of this entity and its proper diagnosis and treatment. The weakness of this article is the heterogeneity of studies used in the literature review. The majority of which are reviews and retrospective case studies. There is an overall scarcity of randomized or prospective trials on this disease process in the literature.

## CONCLUSIONS

This article represents the first comprehensive review that synthesizes information about the pathophysiology, clinical manifestations, and proper diagnostic imaging and treatments associated with radicular pain due to ignored lumbosacral neuroforaminal stenosis. Recent advances in diagnostic imaging and surgical techniques have the potential to enhance the timeliness and durability of the treatment options available to patients. Future directions for foraminal stenosis diagnostic imaging include efforts to develop the nascent field of computerized mapping to reliably quantify stenosis and its effect on the exiting nerve root and associated dorsal root ganglia. This article outlines the current understanding of neuroforaminal stenosis and hopes to stress the importance of addressing it in the index surgery in addition to considering it in the failed back surgery. Future studies should fill the gap in further understanding the causes for failures in lumbar spinal stenosis and consider neuroforaminal stenosis as a cause.

### Conflict of Interest

None

### Ethical Statement

No copyrighted materials or protected health information are reproduced in this manuscript. The submitted manuscript fulfills all IRB research ethics and compliance requirements. Furthermore, the foregoing manuscript does not contain any information pertaining to medical devices or proprietary efforts. Please note that the contents of the foregoing manuscript have not been published in any other sources and are not being submitted to any other publication venues for consideration.
